# Identification of potential chemical compounds enhancing generation of enucleated cells from immortalized human erythroid cell lines

**DOI:** 10.1038/s42003-021-02202-1

**Published:** 2021-06-03

**Authors:** Svetlana Soboleva, Ryo Kurita, Fredrik Ek, Hugo Åkerstrand, Rita Silvério-Alves, Roger Olsson, Yukio Nakamura, Kenichi Miharada

**Affiliations:** 1 Division of Molecular Medicine and Gene Therapy, Lund Stem Cell Center, Lund University, Lund, Sweden; 2 Department of Research and Development, Central Blood Institute, Japanese Red Cross Society, Tokyo, Japan; 3 Chemical Biology and Therapeutics, Department of Experimental Medical Science, Lund University, Lund, Sweden; 4 Division of Molecular Hematology, Lund Stem Cell Center, Lund University, Lund, Sweden; 5 Wallenberg Center for Molecular Medicine, Lund University, Lund, Sweden; 6 Cell Engineering Division, RIKEN BioResource Research Center, Tsukuba, Ibaraki Japan; 7 International Research Center for Medical Sciences, Kumamoto University, Kumamoto, Japan

**Keywords:** High-throughput screening, Cell division, Extracellular signalling molecules, Regenerative medicine, Tissue engineering

## Abstract

Immortalized erythroid cell lines are expected to be a promising source of ex vivo manufactured red blood cells (RBCs), however the induction of enucleation in these cell lines is inefficient at present. We utilized an imaging-based high-throughput system to identify chemical compounds that trigger enucleation of human erythroid cell lines. Among >3,300 compounds, we identified multiple histone deacetylase inhibitors (HDACi) inducing enucleated cells from the cell line, although an increase in membrane fragility of enucleated cells was observed. Gene expression profiling revealed that HDACi treatment increased the expression of cytoskeletal genes, while an erythroid-specific cell membrane protein, *SPTA1*, was significantly down-regulated. Restoration of *SPTA1* expression using CRISPR-activation partially rescued the fragility of cells and thereby improved the enucleation efficiency. Our observations provide a potential solution for the generation of mature cells from erythroid cell lines, contributing to the future realization of the use of immortalized cell lines for transfusion therapies.

## Abstract (web summary)

In an imaging-based screen of >3,300 compounds compounds, Soboleva et al identify HDAC inhibitors as mediators of erythroid cell enucleation. They further show that the erythroid-specific cell membrane protein, SPTA1, is downregulated in HDAC inhibited cells and that restoration of SPTA1 expression using CRISPR-activation partially rescues the fragility of cells, improving enucleation efficiency.

## Introduction

Ex vivo production of functional red blood cells (RBCs) is a potential solution to the current shortage of blood supply in donor-dependent transfusion therapies^1,2^. In addition, this is an attractive method of RBC generation, as the production process can be operated under strict quality control, minimizing the risk of infection originating from donated blood. To date, different types of cell sources have been considered as the starting material for ex vivo RBC production. Primary CD34^+^ hematopoietic stem/progenitor cells isolated from umbilical cord blood (UBC) have been used to establish in vitro culture conditions that can efficiently generate enucleated erythrocytes^3–5^. However, due to their limited growth capacity, primary cells (including CD34^+^ cells) may not be an efficient source to provide the large number of RBCs required for transfusion^6,7^. Considering this requirement of high cell number, cells equipped with an unlimited growth capacity may be more suitable sources. Human embryonic stem (ES) cells and induced pluripotent stem (iPS) cells have been considered as alternative starting materials for the large-scale generation of RBCs in vitro, as these cells can be cultured semi-infinitely^8^. Utilizing iPS cells to generate RBCs has an additional value, as they can be generated from cells originating from patients with rare blood types. Although many approaches generating RBCs from human ES/iPS cells have been reported, the efficiency of these methods is not at a sufficient level^9–11^. Considering that ES/iPS cells are maintained at the pluripotent stage, differentiation towards mature erythroid cells requires lengthy and complicated protocols, as well as a high cost. In addition, the efficiency of enucleation is poor, which severely reduces the yield of mature RBCs. Immortalized human erythroid cell lines are expected to be an alternative source for ex vivo production of RBCs, as these cells are already committed to the erythroid lineage and possess a limitless growth capacity. We have previously established immortalized erythroid cell lines derived from human UCB-derived CD34^+^ cells (HUDEP) and human iPS cells (HiDEP), by ectopically overexpressing human papilloma virus E6/E7 gene (*HPV-E6/E7*)^12^. HUDEP/HiDEP sustain infinite growth capacity, express erythroid-specific cell surface markers (e.g., Glycophorin-A), and produce functional hemoglobin^12^. However, similar to erythroid cells differentiated from ES/iPS cells, these cells do not efficiently enucleate and exhibit a high level of cell death upon the induction of differentiation. Other than HUDEP/HiDEP, several groups have established immortalized human erythroid cell lines from UCB cells and iPS cells using other combinations of genes^13–15^. These cell lines, however, also have the issue of poor enucleation efficiency. Thus, developing an efficient method to induce enucleation is one of the biggest hurdles before the utilization of such immortalized cells for large-scale ex vivo RBC production is possible and therefore needs to be addressed.

Enucleation is a dynamic, rapid, and strictly regulated process^16,17^. It has been proposed that enucleation is similar to asymmetrical division, in which microtubules assist the establishment of cell polarity and Rac GTPase, through actin polymerization, controls the formation of the actomyosin ring separating the nucleus and reticulocyte^18–20^. These findings potentially explain how enucleation of normal erythroid cells is regulated. Immortalized cell lines, however, may have distinct regulative mechanisms controlling enucleation. Thus, discovering key gene(s) and pathway(s) involved in the enucleation process of immortalized cell lines may need unbiased approaches.

A chemical compound screen is a comprehensive and direct approach to discover new regulators or pathways in target cells. In this study, we employed an imaging-based high-throughput screening system, combined with two distinct DNA dyes to capture morphological changes of the immortalized erythroid cell lines. Among >3300 chemical compounds, we identified multiple histone deacetylase (HDAC) inhibitors (HDACi) that largely increased the frequency of enucleated cells. Gene expression profiling revealed that HDACi treatment led to the induction of various cytoskeletal genes including kinesins. In addition, one critical membrane component, *SPTA1*, was significantly downregulated, resulting in the increased fragility of enucleated cells. Restoration of *SPTA1* using clustered regularly interspaced short palindromic repeat (CRISPR) activation (CRISPRa) significantly improved viability of the enucleated cells and, as a consequence, increased enucleation efficiency. Our findings propose a potential method of increasing the generation of enucleated cells from immortalized erythroid cell lines, which is expected to contribute to enable large-scale ex vivo RBC production.

## Results

### The identification of candidate chemical compounds inducing putative enucleation through a large-scale Cellomics ArrayScan screen

**Fig. 1:**
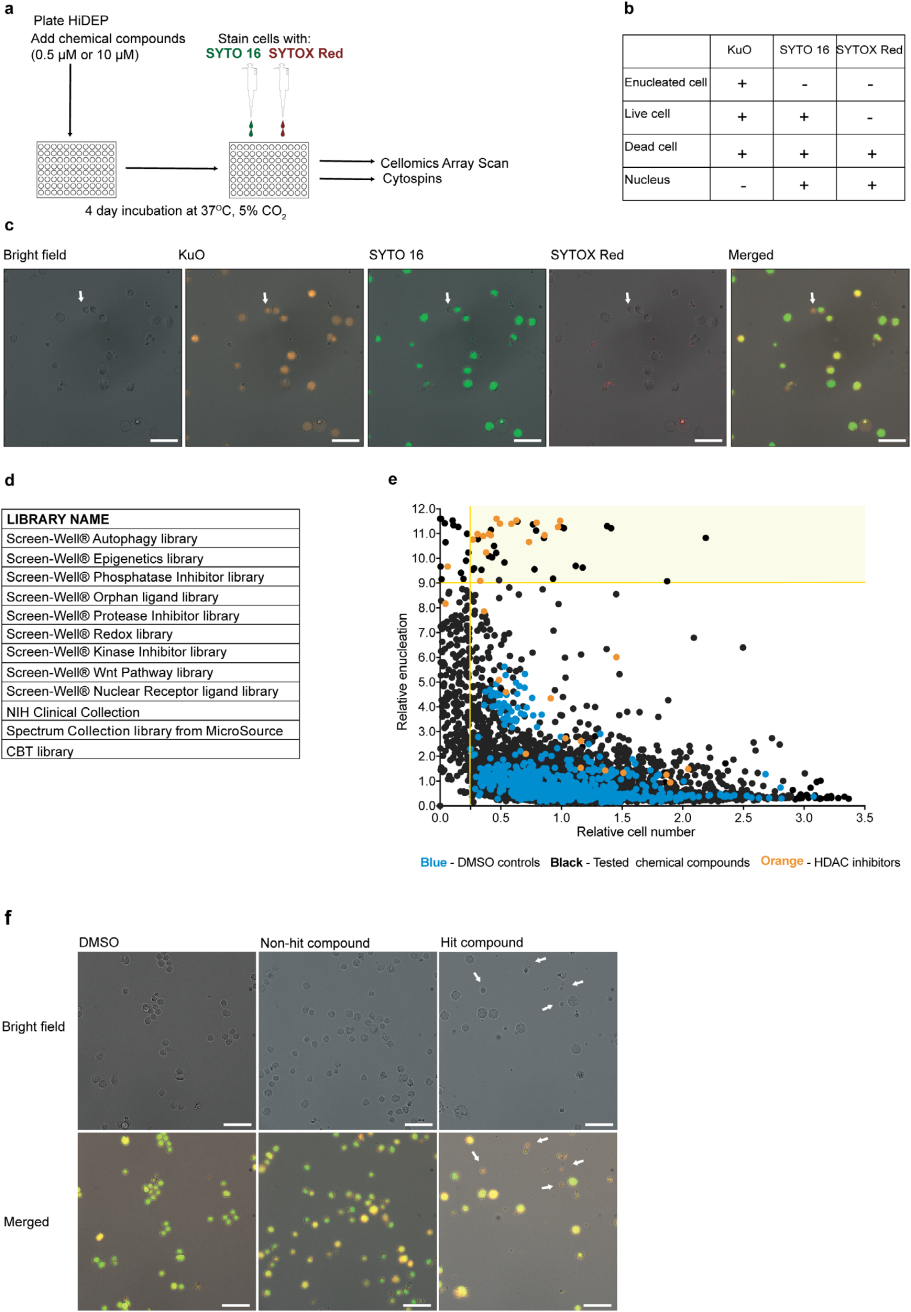
A high-throughput chemical compound screening system using Cellomics ArrayScan. **a** Schematic of experimental procedure. Ten thousand HiDEP-1 cells (HiDEP) per well were cultured with 0.5 μM or 10 μM of chemical compounds dissolved in DMSO for 4 days. Cells were then stained with SYTO 16 and SYTOX Red, and subsequently subjected to Cellomics Arrayscan analysis and cytospin analysis. **b** Theoretically expected output patterns of cell statuses based on the three fluorescent signals. KuO: Kusabira Orange, representing an expression of HPV-E6/E7; SYTO 16: cell permeant dye that stains nuclei of both live and dead cells; SYTOX Red: cell non-permeant dye that stains nuclei of dead cells. **c** Representative fluorescence images of HiDEP stained with two DNA dyes. From left to right: bright-field, Kusabira Orange, SYTO 16, SYTOX Red, and Merged image. The white arrow shows an enucleated cell that is positive for KuO but negative for SYTO 16 and SYTOX Red. Scale bars represent 20 μm. **d** List of chemical compound libraries used in this study. CBT library: in-house novel and nondisclosed chemicals from Chemical Biology and Therapeutics group, Lund University. **e** Result of the compound screening. All chemical compounds were tested at 0.5 μM and 10 μM. The *X*-axis represents relative numbers of KuO^+^ cells and the *Y*-axis represents a relative number of SYTO 16^−^ SYTOX Red^−^ cells. The values are calculated as fold change compared to the mean value of DMSO controls in the same test plate. Light yellow area marks chemical compounds that achieved more than 9.0-fold increase in the enucleation frequency and not <0.25-fold in the total cell number therefore selected as hits. Black dots, tested chemical compounds; blue dots, DMSO controls; orange dots, HDACi. **f** Representative images of cells captured on Cellomics Arrayscan. Bright-field (upper panel) and combinatory staining (Merged, lower panel) of cells treated with DMSO, a representative non-hit compound and one of hit compounds are shown. The white arrows show enucleated cells. Scale bars represent 20 μm.

To perform a quantitative, rapid measurement of enucleation efficiency, we employed the Cellomics^TM^ ArrayScan® system, which captures morphological information and fluorescent signals of cells from multiple wells. The number and frequency of each cell type are determined by the combination of fluorescent signals. HiDEP-1 (HiDEP) were cultured with chemical compounds for 4 days and then subjected to the Cellomics ArrayScan assay (Fig. 1a). HiDEP-1 was selected, as they synthesize functional hemoglobin even during maintenance conditions, whereas other cell lines need a pre-maturation step to initiate hemoglobin synthesis^12^. To distinguish each cell type based on DNA content and membrane permeability, we utilized two different types of nucleic acid dyes: (i) SYTO 16, a cell permeant nucleic acid dye; and (ii) SYTOX Red, a cell non-permeant nucleic acid dye. In addition, expression of HPV-E6/E7 genes in HiDEP is coupled with Kusabira Orange (KuO)^12^, meaning that all viable HiDEP are positive for KuO. The combination of these three fluorescent signals allows us to distinguish distinct cellular status, as SYTO 16 penetrates both dead and viable cells’ membrane, whereas SYTOX Red stains dead cells and extruded nuclei (Fig. 1b). Based on this principle, enucleated cells are defined as events positive for KuO and negative for both SYTO 16 and SYTOX Red. Enucleation frequency is calculated as the frequency of KuO^+^ SYTO 16^−^ SYTOX Red^−^ events within total KuO^+^ events (Fig. 1b). A test analysis using untreated HiDEP showed all types of events, with the vast majority of cells being double positive for KuO and SYTO 16 (Fig. 1c). As expected, very few enucleated cells and dead cells were found according to our definition, as HiDEP have a minimum frequency of spontaneous enucleation under maintenance conditions^12^.

Using this method, we screened 3327 chemical compounds from multiple libraries (Fig. 1d) at two concentrations (0.5 μM or 10 μM). We observed negligible difference in both cell number and enucleation frequency compared to dimethyl sulfoxide (DMSO) controls in the vast majority of tested compounds, although several chemical compounds increased the number of putative enucleated cells (Fig. 1e, f). Treatment with most of these compounds resulted in a modest decrease in the total cell number compared to the DMSO control, presumably, because cell growth was blocked/terminated upon treatment with effective compounds, whereas cells treated with non-effective compounds and DMSO continued cell division during the culture period. Several compounds induced a severe decrease in the cell number, this effect considered to be due to potential cytotoxicity (Fig. 1e). On the basis of these considerations, compounds achieving >9.0-fold increase in the enucleation frequency but not <0.25-fold in the total cell number were selected as hit compounds (Fig. 1e and Table 1).

**Table 1:** List of hit compounds.

Name	Concentration	Enucleation	Cell number
Trichostatin A	0.5 μM	11.60	0.46
Trichostatin A	10 μM	11.53	0.63
Vorinostat (SAHA)	10 μM	11.52	0.99
Cantharidic acid	10 μM	11.49	0.64
CI-994	10 μM	11.44	0.80
BML-281	10 μM	11.40	0.49
NCH-51	10 μM	11.40	0.59
Fluorescein	10 μM	11.37	0.77
d-Isoleucine	10 μM	11.30	1.38
Fluoro-SAHA	10 μM	11.26	0.97
l-Cysteine sulfinic acid	10 μM	11.26	1.01
d-Histidine	10 μM	11.22	1.02
d-Aspartic acid	10 μM	11.22	1.41
Cantharidin	10 μM	11.14	0.42
*N*-Acetyltryptamine	10 μM	11.13	0.79
Apicidin	10 μM	10.98	0.40
M344	0.5 μM	10.98	0.31
Scriptaid	10 μM	10.94	0.42
Vorinostat (SAHA)	0.5 μM	10.93	0.86
Apicidin	0.5 μM	10.90	0.35
d-Histidine	0.5 μM	10.83	2.19
5-Methoxytryptamine	10 μM	10.82	0.86
*n*-Octyl caffeate	10 μM	10.78	0.31
Suberoyl bis-hydroxamic acid	10 μM	10.76	0.27
M344	10 μM	10.66	0.73
9,10-Phenanthrenequinone	0.5 μM	10.50	0.44
Oxamflatin	10 μM	10.24	0.38
Tanshinone IIA	10 μM	10.22	0.46
PYRROMYCIN	10 μM	10.09	0.40
9,10-Phenanthrenequinone	10 μM	10.04	0.43
U74389G maleate	10 μM	9.84	0.53
Myosmine	10 μM	9.70	1.12
Tryptamine·HCl	10 μM	9.63	1.18
EPIRUBICIN HYDROCHLORIDE	10 μM	9.60	0.28
C27H22Cl2N4	10 μM	9.56	0.78
C16H26O5	10 μM	9.54	0.34
Foxy-5	0.5 μM	9.18	0.93
C43H55N5O7.H2O4S	0.5 μM	9.12	0.49
BML-210	10 μM	9.09	0.33
d-Aspartic acid	0.5 μM	9.07	1.87

The name of compounds, used concentration, enucleation efficiency, and viability (fold change) at day 4 are shown.

### Treatment with a series of HDACi can increase putative enucleated cells

**Fig. 2:**
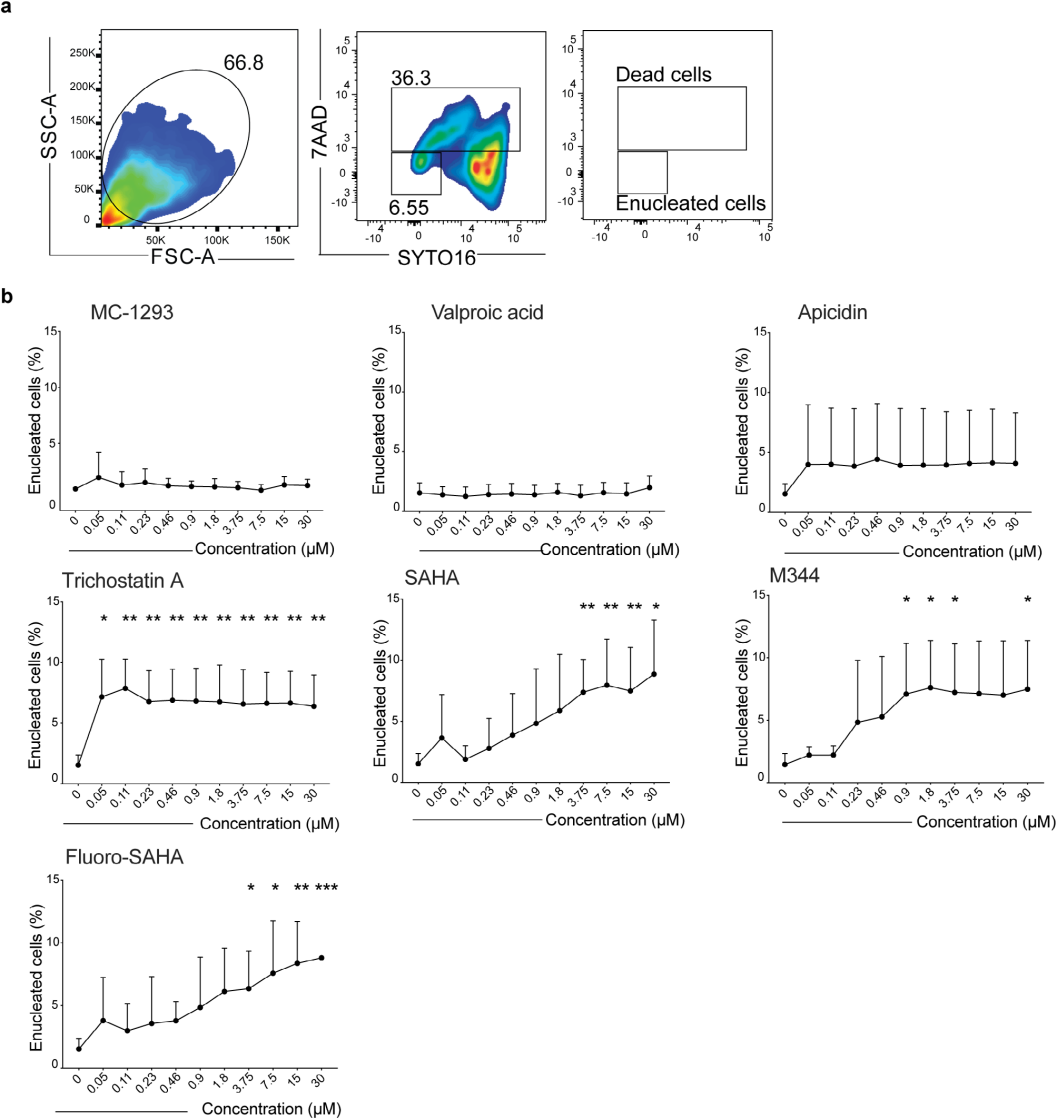
Validation of the effect of HDACi on induction of enucleation using flow cytometry. **a** Representative FACS profiles of HiDEP stained with 7AAD and SYTO 16. Cells treated with DMSO (left) and Fluoro-SAHA (right) are shown. **b** Dose response of HiDEP to selected HDACi. Enucleation frequencies of HiDEP treated with different concentrations of HDACi at day 1 are shown. Mean ± SD, *n* = 7. Significance was determined using one-way ANOVA with Dunnett’s multiple comparisons test. *p < 0.05, **p < 0.01, ***p < 0.001.

Among the *hit* compounds we found multiple (12 out of 16) HDACi (Table 1). HDAC are posttranscriptional regulators that remove acetyl groups from amino-acid residues on histone tails, allowing the affected histones to tightly wrap DNA and repress gene translation^21^. HDACi are chemical compounds that inhibit the enzymatic activity of HDAC, which dissociate histone proteins from DNA, thereby making genes more accessible for transcription^22,23^. Implications of HDAC and effects of HDACi on erythropoiesis have been previously described^24–27^; however, these observations were made from studies of primary erythroid cells in which HDACi were described as negative effectors of enucleation. To validate the function of compounds identified in our screen, HiDEP were treated with selected HDACi at multiple concentrations and enucleation efficiency was analyzed using flow cytometry. As previously reported^28^, enucleated cells were found in FSC^low^SYTO 16^−^7AAD^−^ population, whereas intact nuclei were mainly contained in FSC^low^SYTO 16^+^7AAD^−^ population. Vast majority of the cells contained in these populations were positive for Glycophorin-A, indicating that those cells/nuclei were surrounded by cell membrane. SYTO 16^+^7AAD^+^ populations contained both intact nuclei and dead cells (Supplementary Fig. 1a, b). To calculate the frequency of enucleated cells within these different cell types, enucleation efficiency was defined by the percentage of SYTO 16^−^7AAD^−^ cells in both FSC^high^ and FSC^low^ cells (Fig. 2a). We could confirm that treatment with a number of HDACi that exhibited increased enucleation ratio in the initial screening also resulted in higher frequencies of putative enucleated cells. Each HDACi had a distinct optimal concentration and duration of the activity, which may be due to a different half-life and affinity for their target(s) (Fig. 2b). None of these compounds showed a significant reduction in the viability of treated cells on the basis of 7-Amino-Actinomycin-D (7AAD) staining (Supplementary Fig. 1c). We also tested to treat HUDEP-2 that is derived from human UBC CD34^+^ cells and express adult globin^12^, and saw the similar response upon Fluoro-SAHA (FS) treatment (Supplementary Fig. 1d). In contrast, an erythro-leukemia cell line, K562, did not respond to FS (Supplementary Fig. 1e). Furthermore, primary erythroid cells induced from UCB-derived CD34^+^ cells^5^ were treated with FS. Erythroid cells after 6-day culture responded to the treatment, but many of them were collapsed. In contrast, erythroid cells after 10-day culture showed only mild response upon the treatment (Supplementary Fig. 1f). These results indicate that the effect of selected HDACi is not general but selective.

In addition to HDACi, other 24 compounds were also identified as *hit* compounds (Table 1); however, flow cytometry analyses did not confirm the effect of these compounds on the enhancement of enucleation. Based on these evaluations, we selected two top-hit compounds, FS and M344, for further studies.

### Motional and morphological analyses confirm enucleation induced by HDACi

**Fig. 3:**
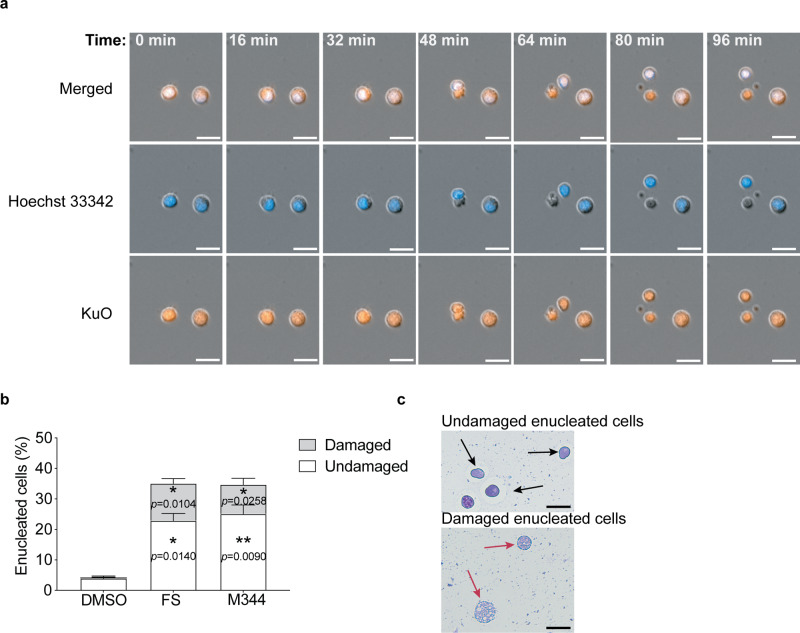
Motional and morphological confirmation of enucleation induced upon HDACi treatment. **a** Captured images of enucleating HiDEP from the time-lapse imaging. HiDEP were stained with Hoechst 33342, plated into 96-well plates, incubated with 15 μM Fluoro-SAHA, and visualized in the Celldiscoverer 7 system. Images of a representative enucleating cells taken every 16 min are shown. Scale bars represent 20 μm. **b** Frequency of enucleated cells after Fluoro-SAHA treatment. Undamaged (intact) and damaged enucleating cells were manually counted on 45–50 fields from cytospin slides. Mean values of enucleation frequency in 3 slides ± SD are shown. Significance was determined using one-way ANOVA with Dunnett’s multiple comparisons test. **c** Representative May–Grünwald–Giemsa staining of cytospin slides of HiDEP cells treated with DMSO or Fluoro-SAHA. The black arrows show undamaged enucleated cells and red arrows indicate damaged cells. Scale bars represent 25 μm. *p < 0.05, **p < 0.01.

To ensure that cells without nuclei were a result of enucleation, in which the nucleus is pushed out from the cell, we monitored the motion of HiDEP treated with FS, using Celldiscoverer 7 live-cell imaging system. Time-lapse imaging visualized HiDEP undergoing extrusion of the nucleus as typically observed during the enucleation of primary erythroblasts (Fig. 3a and Supplementary Movie 1). In addition to standard enucleation, we also observed several HiDEP causing “rupture” events that produced multiple small-enucleated cells (Supplementary Movie 2). The rupture event may be distinct from typical apoptosis, as the main large cell body still remained even after small cell fragments were produced. In addition, the nucleus of a cell undergoing apoptosis is normally divided into small fragments and some apoptotic bodies contain nuclear contents; however, we observe that the small-enucleated cells did not contain nucleic acid (Supplementary Movie 2). Taking this into consideration, the small-enucleated cells may be microparticles or extracellular vesicles caused by cytoplasmic protrusion caused under both apoptotic and non-apoptotic conditions^29^. These findings support that FS has the potential to induce enucleation of HiDEP. There are however two types of cellular responses upon FS treatment and the rupture event may be distinct from standard enucleation seen in primary erythroid cells.

To quantify enucleation frequency, morphological observation would be more accurate to count enucleated cells. We therefore performed the cytospin assay after FS treatment and manually counted the number of enucleated cells. FS treatment significantly increased the frequency of enucleated cells, as ~23% of cells were enucleated cells, whereas DMSO treatment contained only 4% of enucleated cells (Fig. 3b). However, we noticed that many of the enucleated cells were fragile and their cell membranes were destroyed (Fig. 3b, c).

These results suggest that FS/M344 treatment leads to enhanced enucleation, while produced cells are damaged, and known culture methods are not capable to improve the viability.

### HDACi treatment leads to differentiation of HiDEP

**Fig. 4:**
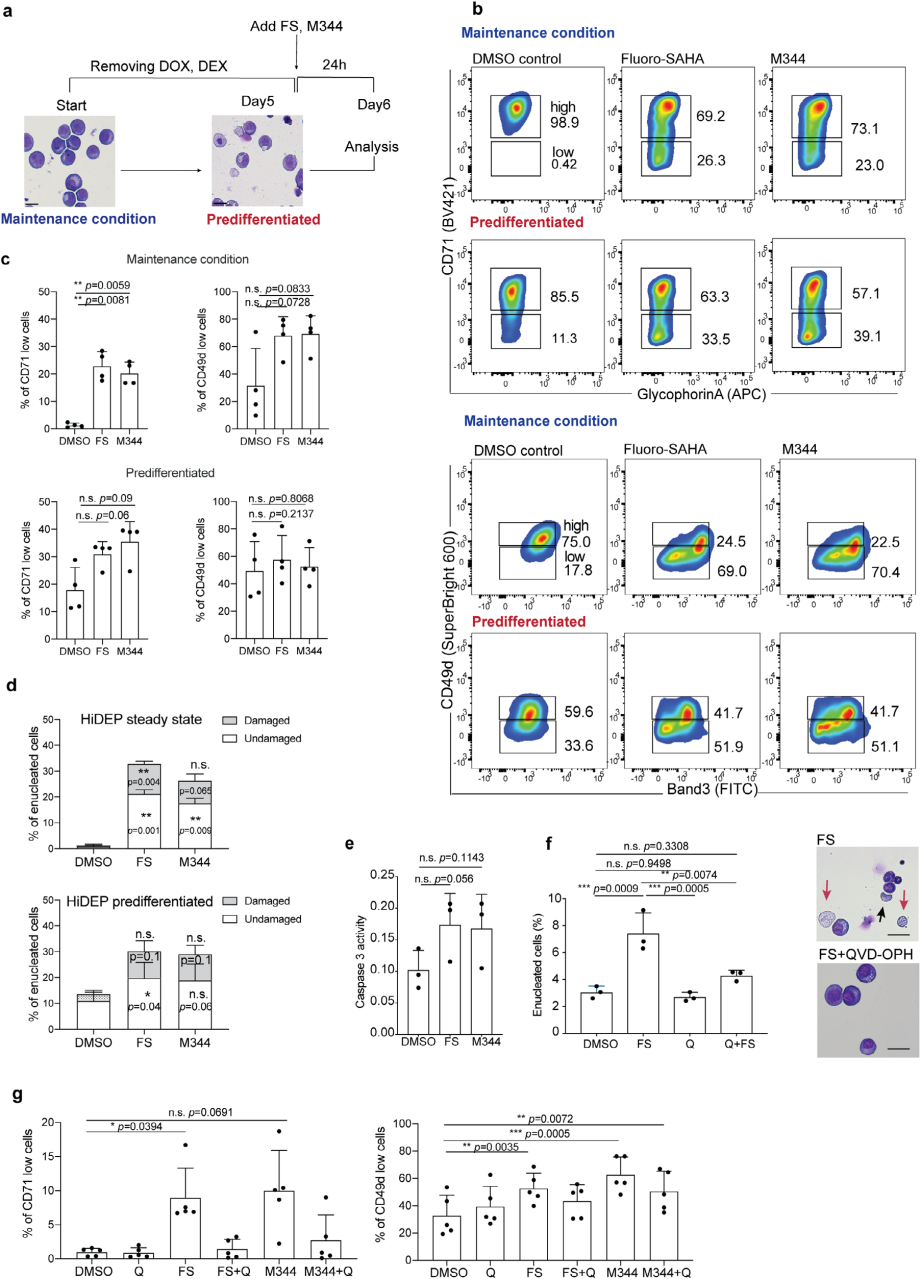
Treatment with HDACi leads to alterations of cell surface phenotype of HiDEP. **a** Schematic of experimental procedure. HiDEP were treated with FS or M344 for 24 h with or without 5-day pre-differentiation. The cells were then subjected to flow cytometry analyses. **b**, **c** Flow cytometry analyses for cell surface expression of representative erythroid cell surface markers. Representative FACS profiles (**b**) and summarized graphs (**c**) are shown. Mean ± SD, *n* = 4. Significance was determined using one-way ANOVA with Dunnett’s multiple comparisons test. **d** Frequency of enucleated cells after Fluoro-SAHA treatment with or without pre-differentiation treatment. *n* = 3. Significance was determined using one-way ANOVA with Dunnett’s multiple comparisons test. **e** Measurement of enzymatic activity of caspase-3 in HiDEP treated with DMSO, Fluoro-SAHA (FS), or M344. Mean ± SD, *n* = 3. Significance was determined using paired *t*-test. **f** Effects of caspase inhibition on enucleation of HiDEP upon HDACi treatment. HiDEP were treated with 50 μM of a pan-caspase inhibitor, QVD-OPH, with or without 15 μM of Fluoro-SAHA. Frequency of enucleated cells was analyzed using flow cytometry. FS, Fluoro-SAHA; Q, QVD-OPH. Mean ± SD, *n* = 3. Significance was determined using one-way ANOVA with Tukey’s multiple comparisons test. **g** Cell surface expression of representative erythroid cell surface markers on HiDEP treated with HDACi with or without QVD-OPH treatment. *n* = 5. FS, Fluoro-SAHA; Q, QVD-OPH. Mean ± SD, *n* = 5. Significance was determined using one-way ANOVA with Dunnett’s multiple comparisons test. *p < 0.01, **p < 0.01, ***p < 0.001.

HDAC have been implicated in differentiation and maturation of primary erythroid cells^24–27^. As the increase in enucleated cells upon HDACi might be the consequence of enhanced differentiation but not directly triggered, we next asked whether pre-differentiation of HiDEP further enhances enucleation or rather inhibits the response. HiDEP were treated with HDACi under the maintenance condition or cultured in the differentiation condition^12^ for 5 days and then treated with HDACi, and the flow cytometry analyses were employed to analyze erythroid differentiation and enucleation (Fig. 4a). Analyses of representative cell surface markers for erythroid cells observed that the expression levels of CD49d and CD71 significantly decreased upon pre-differentiation treatment (Fig. 4b, c), which is seen during the differentiation from polychromatic erythroblasts to orthochromatic erythroblasts^30,31^. FS and M344 treatment also resulted in the reduction of these markers on HiDEP when they were treated under maintenance condition; however, after the pre-differentiation the reduction of CD49d and CD71 expression was moderated (Fig. 4b, c). Of note, HDACi treatment of pre-differentiated cells still showed a trend of the increase in the number of enucleated cells but the difference was insignificant (Fig. 4d), indicating that enucleation might be induced separately from differentiation but was largely dependent on the differentiation process.

Caspases are key enzymes playing a major role in apoptosis, which, in particular caspase-3, have also been implicated in proliferation, differentiation, and maturation of primary erythroid cells^32,33^. In contrast, Yoshida et al.^28^ has demonstrated that enucleation can be induced independent of Caspase-3 activity. We found that caspase-3 was active in HiDEP and tended to increase upon FS/M344 treatment (Fig. 4e). We therefore tried to improve cell viability by adding QVD-OPH, a pan-caspase inhibitor, during the FS treatment. However, rescue of cell fragility was modest, while changes of cell surface markers and the increase in the number of enucleated cells were severely inhibited (Fig. 4f, g). These findings indicate that caspase activity is necessary for differentiation and enucleation of HiDEP induced by HDACi, and therefore caspase inhibition is not instrumental in saving the intactness of enucleated cells.

### FS and M344 mainly target Class I HDAC in HiDEP

**Fig. 5:**
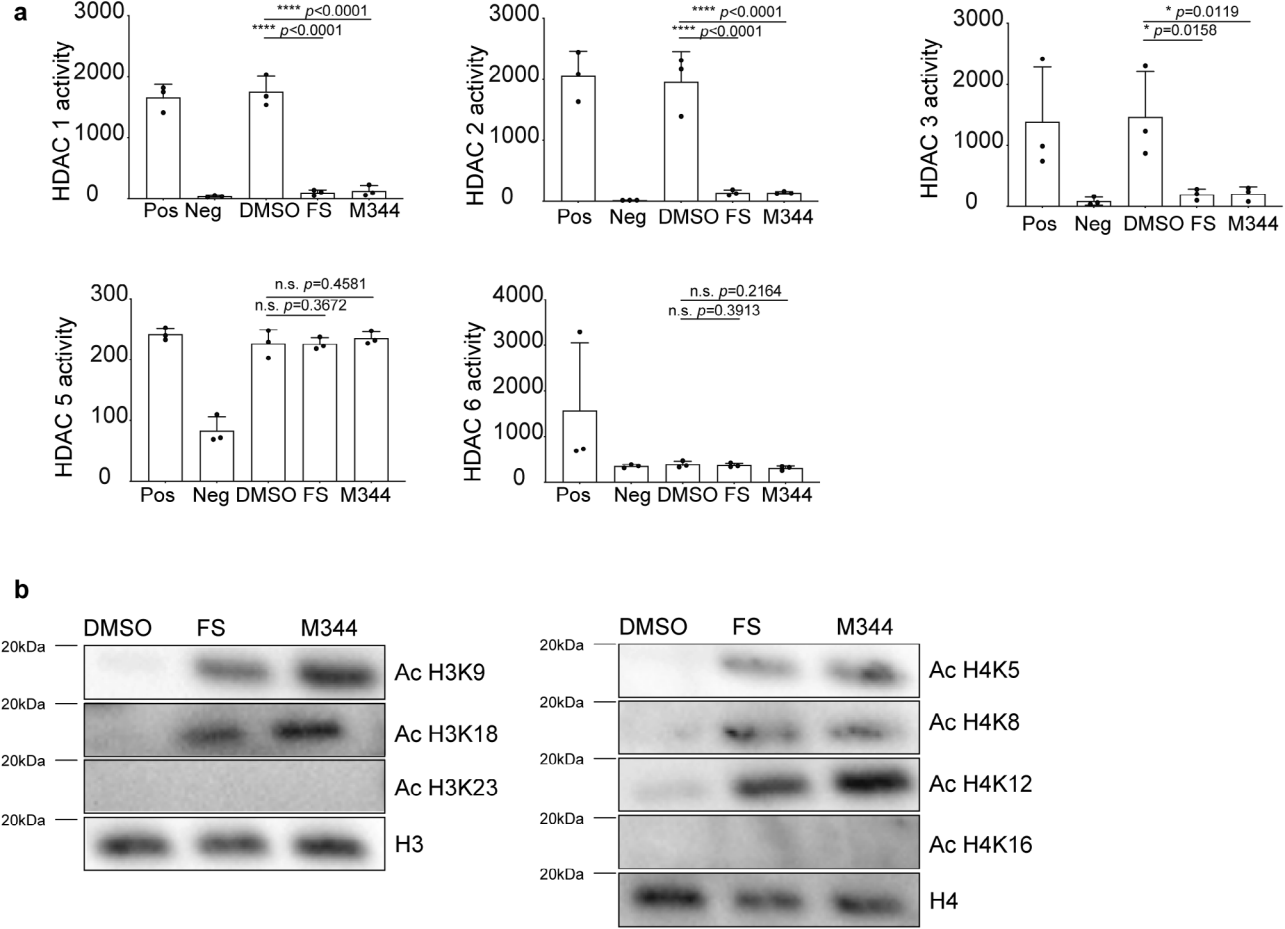
FS and M344 mainly target Class I HDAC in HiDEP. **a** Measurement of enzymatic activity of HDAC in HiDEP treated with DMSO or FS, or M344. Positive control (Pos) and negative control (Neg) contained in the assay kit are also shown. Mean ± SD, *n* = 3. Significance was determined using one-way ANOVA with Dunnett’s multiple comparisons test. **b** Western blot analysis of acetylation states of histone H3 and H4 in HiDEP treated with FS or M344. Total histone H3 and histone H4 are used as reference controls. One of three replicated experiments is shown. *p < 0.05, ****p < 0.0001.

As the HDAC are categorized to five subgroups (Class) and each HDACi could target a different range of HDAC^23^, we next studied which HDAC were targeted by FS and M344. Quantitative reverse-transcriptase PCR (qRT-PCR) analysis observed that in HiDEP *HDAC1*, *HDAC2*, *HDAC3*, *HDAC5*, and *HDAC6* are expressed (Supplementary Fig. 2). To specify target HDAC of FS and M344, enzymatic activity of selected HDAC was analyzed. The assay showed that activities of HDAC1, HDAC2, and HDAC3 (all belong to Class I) were significantly reduced within 60 min, whereas HDAC5 (Class IIa) and HDAC6 (Class IIb) did not show the difference in their activity (Fig. 5a). As inhibition of HDAC activity is expected to lead to acetylation of histone proteins, we next analyzed global histone acetylation using antibodies targeting specific lysine residues, in particular histone H3 and H4, which have been implicated in erythroid maturation^34^. Treatment with both FS and M344 resulted in clear acetylation of histone H3 and H4 at a broad range of lysine residues, including H3K9, H3K18, H4K5, H4K8, and H4K12, whereas H3K23 and H4K16 acetylation was not detected (Fig. 5b and Supplementary Fig. 3). These observations imply that a drastic and a wide range of gene expression change could be induced upon FS and M344 treatment.

### Microarray analysis reveals key genes involved in the differentiation of HiDEP

**Fig. 6:**
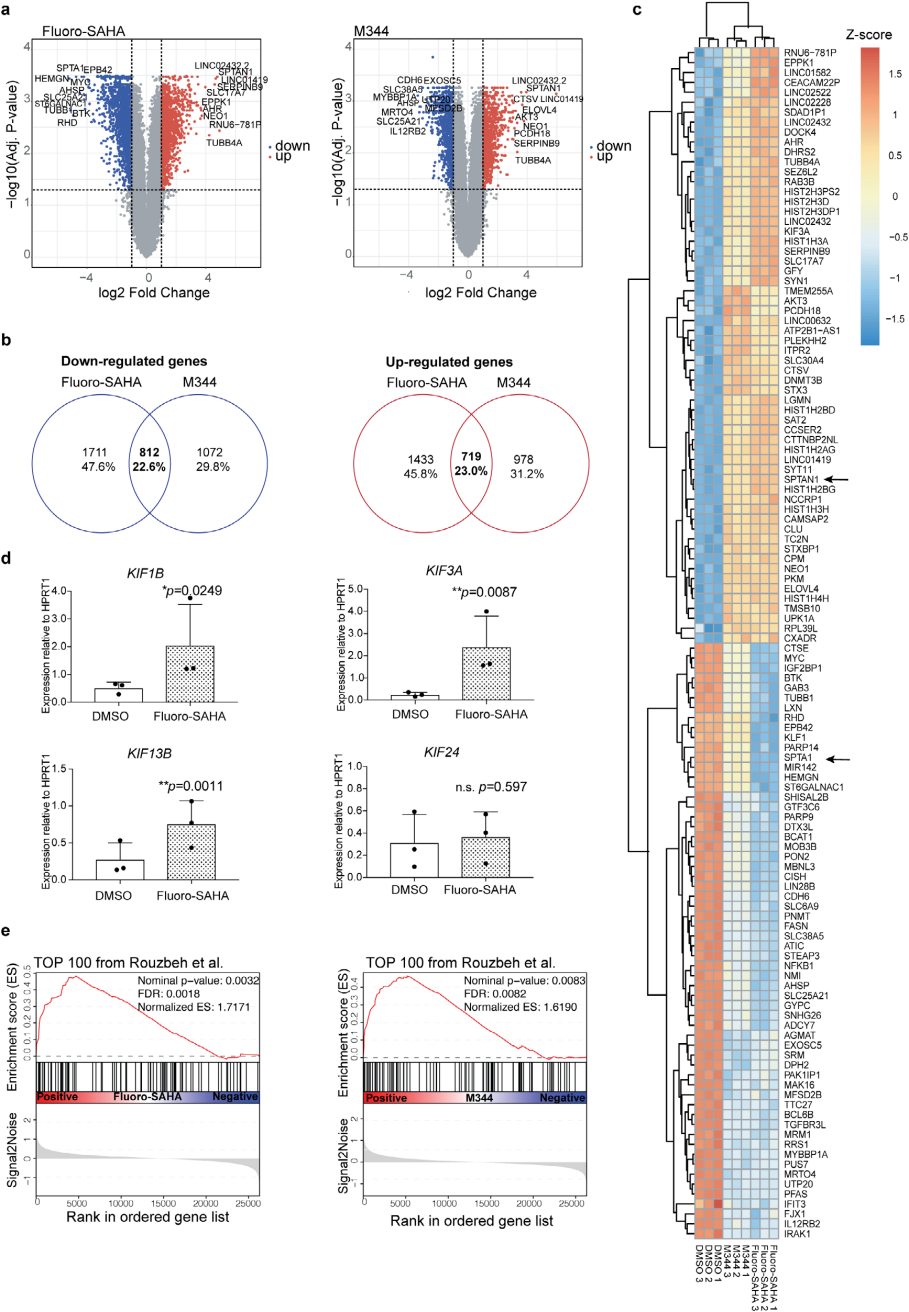
Gene expression profiles of FS- and M344-treated HiDEP cells. **a** Volcano plots of the microarray data comparing gene expression profiles of HiDEP cells treated with Fluoro-SAHA and M344 for 24 h. **b** Venn diagrams showing overlap of genes down- and upregulated upon Fluoro-SAHA and M344 treatment. The number of genes and frequency within the whole altered genes are shown. **c** A heatmap of 60 selected genes that are commonly up- and downregulated upon both Fluoro-SAHA and M344 treatment. **d** qRT-PCR analysis for expression of kinesin genes in HiDEP cells treated with DMSO or Fluoro-SAHA. The expression levels are normalized to *HPRT1*. Mean ± SD, *n* = 3. Significance was determined using paired *t*-test. **e** Gene set enrichment analysis (GSEA) of the microarray data. Enrichment of top 100 genes that are upregulated in erythroblasts differentiated from human iPS cells^37^ are shown. *p < 0.05, **p < 0.01.

To identify key genes involved in the differentiation and leading to the cellular fragility of HiDEP upon HDACi treatment, a microarray analysis was performed to compare gene expression profiles of HiDEP treated with FS, M344, or DMSO (Fig. 6a). Upon FS treatment 2152 genes were significantly upregulated and 2523 genes were downregulated compared to DMSO-treated cells, whereas M344 treatment upregulated 1697 genes and downregulated 1884 genes. Among those genes, 719 genes (23.0%) and 812 genes (22.6%) were commonly up- or downregulated by FS and M344 (Fig. 6b). The top 60 genes commonly upregulated upon FS and M344 included multiple histone-component genes (e.g., *HIST2H3D*, *HIST1H3A*, *HIST1H2BD*), representing the effects of histone modifications by HDACi (Fig. 6c). Commonly upregulated genes also contained a variety of cytoskeletal genes, including kinesins (e.g., *KIF3A* and *KIF1B*), molecular motors previously implicated in the enucleation process^20,35^, whose induction was confirmed by qRT-PCR analysis (Fig. 6b–d). In contrast, downregulated genes included *MYC*, downregulation of which is required during enucleation of murine fetal liver erythroid progenitor cells^36^. We compared overall gene expression changes with the data by Rouzbeh et al.^37^ showing molecular signatures upregulated upon differentiation from human ES cell-derived embryoid body into erythroblast and found that top 100 genes induced during the differentiation were significantly enriched in both FS- and M344-treated HiDEP (Fig. 6e). This observation indicates that gene expression change upon FS/M344 treatment is similar to in vitro erythroid differentiation process of infinite cell sources.

### Forced activation of *SPTA1* using CRISPRa improves viability and enucleation efficiency of HiDEP upon HDACi treatment

**Fig. 7:**
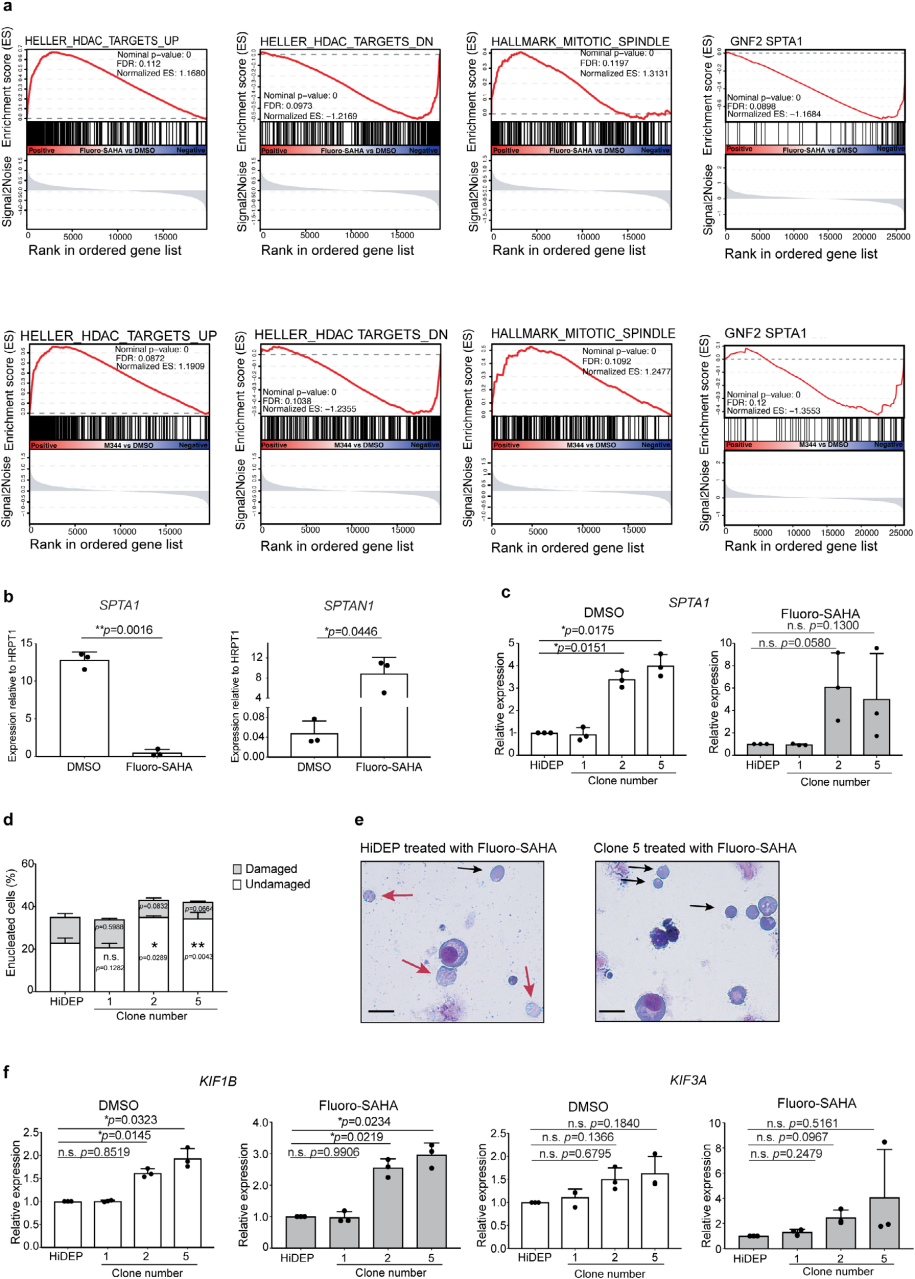
Restoration of *SPTA1* expression partially alleviates fragility of enucleated cells. **a** Gene set enrichment analysis (GSEA) of the microarray data. Gene signatures enriched in Fluoro-SAHA-treated cells (upper panel) and M344-treated cells (lower panel) are shown. **b** qRT-PCR analysis of *SPTA1* and *SPTAN1* expression in HiDEP treated with DMSO or Fluoro-SAHA. Mean ± SD, *n* = 3. Significance was determined using paired *t*-test. **c** Relative expression of *SPTA1* in HiDEP and CRISPRa-lines analyzed by qRT-PCR. The expression levels are normalized to *HPRT1*. Mean ± SD, *n* = 3. Significance was determined using one-way ANOVA with Dunnett’s multiple comparisons test. **d** Frequency of enucleated cells in HiDEP and CRISPRa-lines treated with Fluoro-SAHA. Intact enucleated cells (undamaged) and damaged enucleated cells were manually counted on cytospin slides. Mean ± SD, *n* = 3. Significance was determined using one-way ANOVA with Dunnett’s multiple comparisons test. **e** Representative May–Grünwald–Giemsa staining of cytospin slides of HiDEP cells activating *SPTA1* (clone #5) treated with DMSO or Fluoro-SAHA. **f** Relative expression of *KIF1B* and *KIF3A* in HiDEP and CRISPRa-lines analyzed by qRT-PCR. The expression levels are normalized to *HPRT1*. Mean ± SD, *n* = 3. Significance was determined using one-way ANOVA with Dunnett’s multiple comparisons test. *p < 0.05, **p < 0.01.

To discover key pathways involved in the FS/M344-induced enucleation, gene set enrichment analysis (GSEA) was performed. GSEA of the microarray data confirmed the function of two compounds inhibiting the effect of HDAC (Fig. 7a). In addition, a mitotic spindle signature was enriched in both FS- and M344-treated cells compared to DMSO-treated control cells, which points to the ability of these HDACi to trigger cytoskeletal rearrangement. Of note, we found a reduction of the SPTA1-related gene signature (Fig. 7a). Erythroid-specific α-spectrin (*SPTA1*, also known as α1 spectrin), one of main components of the RBC membrane, was significantly downregulated, whereas non-erythrocytic α-spectrin (*SPTAN1*, α2 spectrin) was highly and abnormally induced upon both FS and M344 treatment (Fig. 6c). Spectrins are cytoskeletal proteins existing on the intracellular side of RBC membranes and form complexes with other cytoskeletal proteins, which serve to maintain membrane integrity and structure^38^. In erythrocytes, α1 (SPTA1) and β1 spectrins uniquely dimerize and bind to other components of erythroid membranes such as ankyrin, protein 4.1R, and Band 3^38,39^, but SPTAN1 is normally not expressed in erythroid cells^39,40^. The induction of *SPTAN1* expression may also be a cause of the membrane fragility observed in HiDEP, as SPTAN1 protein is known to be cleaved by caspase-3^41^, presumably resulting in a breakdown of intracellular membrane structures. Importantly, our data indicate that caspase is active in HiDEP (Fig. 4d). The severe reduction of *SPTA1* and abnormal induction of *SPTAN1* were also observed in FS/M344-treated Glycophorin-A^+^ cells derived from UCB CD34^+^ cells (Supplementary Fig. 4a), indicating that the conversion of α-spectrin is a common response upon HDACi treatment. We therefore wondered if the abnormal conversion of α-spectrins could be the cause of the high fragility of the enucleated cells. First, we confirmed the downregulation of *SPTA1* and induction of *SPTAN1* by qRT-PCR. Consistent with the microarray data, the expression of *SPTA1* in FS-treated HiDEP was significantly reduced, whereas *SPTAN1* expression was upregulated (Fig. 7b). We next aimed to improve the viability of HiDEP by restoring SPTA1 expression after HDACi treatment. SPTA1 is a large protein (ca. 280 kDa) and is therefore challenging to overexpress using conventional methods. We therefore took advantage of CRISPRa^42,43^ to induce expression of *SPTA1*. After a selection of cells expressing dCas9 and sgRNA, we confirmed that multiple clones (clone #2 and #5) showed higher expression of *SPTA1* (Fig. 7c). These clones exhibited a lower frequency of damaged cells after FS treatment and, as a consequence, enucleation efficiency was higher than in control HiDEP, whereas a clone that failed to enhance SPTA1 (clone #1) did not observe the difference (Fig. 7d, e). The increased enucleation frequency may not be exclusively due to the alleviated fragility, but also due to enhanced maturation and enucleation action. In fact, higher expression of kinesin genes (*KIF1B* and *KIF3A*) and more decrease of CD49d expression upon FS treatment was detected in the *SPTA1*-activated clones (Fig. 7f and Supplementary Fig. 4b). Although the actual molecular mechanism is unclear at the moment, there might be an unknown regulation driven by spectrin to promote erythroid maturation.

We also asked whether suppression of *SPTAN1* induction could attenuate fragility of the cells through restoration of *SPTA1*. HiDEP-expressing short hairpin RNA against *SPTAN1* significantly lowered *SPTAN1* expression after FS treatment; this, however, did not lead to a higher level of *SPTA1* expression (Supplementary Fig. 4c) and neither an increase in enucleation frequency nor decrease in damaged cells was observed (Supplementary Fig. 4d).

The downregulated genes included not only *SPTA1* but also other cell membrane proteins, e.g., *EPB42*, *GYPC*, and *RHD*. Many of these RBC-membrane genes are regulated by a transcription factor *KLF1*^44,45^. In fact, *KLF1* is one of most significantly downregulated genes in HiDEP upon HDACi treatment (Fig. 6c). We therefore overexpressed *KLF1* in HiDEP using lentiviral vectors, to ask whether sustaining KLF1 expression improves enucleation efficiency and/or cellular fragility. qRT-PCR analysis confirmed that *KLF1* expression in HiDEP was maintained after HDACi treatment (Supplementary Fig. 5a); however, frequency of enucleated cells was not higher than *SPTA1*-enhanced cells (Supplementary Fig. 5b). qRT-PCR analysis revealed that KLF1-overexpressing cells also failed to maintain erythroid cell membrane genes including *SPTA1* (Supplementary Fig. 5c), suggesting that the impact of HDACi treatment on induction/reduction of the target genes is greater than their natural gene regulation.

These findings indicate sustaining SPTA1 expression using artificial approaches may be necessary for enucleated cells derived from immortalized cell lines to keep their cell membrane intact.

## Discussion

Cell lines are often equipped with unique molecular features and distinct gene regulatory networks compared to primary cells of the same origin. Primary erythroid cells terminate cell division when the maturation process begins, whereas HiDEP keep growing even after synthesizing hemoglobin^12^. This fact implies that the induction of differentiation and terminal maturation of such cell lines may require totally different types of trigger(s), challenging to ascertain from previous knowledge and experiences studying primary cells. Unbiased screening of chemical compound libraries is one of strongest approaches to address this question. To enable a high-throughput screening, we established the imaging-based screening method using Cellomics ArrayScan. The screen successfully identified FS and M344, members of HDACi, which significantly increased the number of enucleated cells from HiDEP (Figs. 2b and 3b, and Table 1). Enhancement of enucleation in HiDEP by these HDACi was an unexpected finding, as previous studies have described functions of HDAC as a positive regulator of enucleation^22–25^. However, HDAC controls many types of downstream genes, so diverse effects depending on cell type are understandable. Of note, a previous study that included a chemical compound screening using primary murine erythroblasts also identified HDACi, but as inhibitors of enucleation^46^. This fact supports our idea that regulatory mechanisms of established cell lines could be distinct from that of primary cells.

Microarray analysis revealed that highly upregulated genes upon HDACi treatment include those involved in cytoskeleton, motor proteins, histones, microtubules, etc. (Fig. 6a, c). Some of these genes have previously been implicated in the regulation of enucleation process^18^ and RBC-membrane structure. It has been shown that cell membrane remodeling happens during enucleation and reticulocyte maturation^17,47^, suggesting that HDACi treatment might also support the enucleation process through cell membrane re-organization. However, these alterations, in particular α-spectrin switching (Fig. 5b), were considered rather adverse effect of HDACi treatment for erythroid cells. Spectrins are crucial components for cell membrane assembly and are important for maintaining stability, structure, and shape of the cell membrane^38,39^. RBC membranes, in particular, need to afford both mechanical stability and deformability^41^. Therefore, abnormal composition of cell membranes could be the cause of the cell rupture observed upon HDACi treatment. We could, in fact, improve the viability of HiDEP after HDACi treatment by activating *SPTA1* expression using CRISPRa (Fig. 7e, f). As SPTA1 is a large protein, CRISPRa is one of few choices currently available to increase its expression. However, it appears that the enhanced expression of *SPTA1* based on CRISPRa was still affected by HDACi treatment, still resulting in impaired expression even though it was significantly higher than that of control HiDEP (Fig. 7e, f). This was similar in other erythroid cell membrane genes that are regulated by *KLF1*. Overexpression of KLF1 did not succeed to sustain the expression levels of those genes after HDACi treatment (Supplementary Fig. 5). Given that the expression levels of erythroid cell membrane genes, in particular *SPTA1*, critically and lineally correlates to the stability of the cells, another approach to better maintain expression of such genes would be needed to produce highly stable enucleated cells. High fragility of in vitro manufactured RBC is a common problem for other cell types; our findings may therefore represent the general problem. A recent report has proposed that the origin of the cell lines may be critical, as erythroid cell lines established from the bone marrow instead of UCB and ES/iPS cells are less fragile^15^. In the study, HiDEP have been suggested to have an abnormal distribution of cytoskeletal proteins^15^, which may agree with our present findings on the reason of the fragility of HiDEP. Responses of cell lines to identified HDACi could be an imperative assessment to select better suited cell lines and to further study the molecular mechanism of enucleation in these immortalized cell lines. In our study approximately up to 40% of the cells were enucleated cells. Even though HiDEP is a cell line, a certain proportion of cells do not undergo enucleation upon HDACi treatment, but instead result in cell death. Presumably, there is heterogeneity of cellular status, e.g., cell cycle, which determines the response of the cells to HDACi, and synchronizing the status would be required to achieve even higher efficiency.

In conclusion, our study has provided evidence indicating unique molecular regulation in immortalized cell lines that needs to be considered when these types of cells are utilized as materials in future cell therapies, and a streamlining of the screening using the advanced imaging-based system could be a powerful tool to implement a high-throughput screening. These discoveries will accelerate the time to when immortalized cell lines will be used for the large-scale ex vivo production of RBCs.

## Materials and methods

### Cell line and cell culture

HiDEP-1 (HiDEP), HUDEP-2 (HUDEP), and K562 cell lines were obtained from the RIKEN Cell Bank (Tsukuba, Ibaraki, Japan), which are certified as negative for mycoplasma contamination. HiDEP and HUDEP were maintained in serum-free expansion medium (StemSpan^TM^ SFEM, Stem Cell Technologies) supplemented with 1% penicillin/streptomycin (Invitrogen), 3 IU/ml erythropoietin (EPO), 1 μM dexamethasone (Sigma-Aldrich), and 1 μg/ml doxycycline (Sigma-Aldrich). Cells were maintained in normoxic (21% O_2_) incubator with 5% CO_2_ at 37 °C. K562 was maintained in RPMI 1640 medium supplemented with 10% fetal bovine serum (FBS).

### Primary erythroid cell culture

CD34^+^ cells were isolated from human UCB mononuclear cells using the CD34 MicroBead kit (Miltenyi Biotec) by following the manufacturer’s instruction. One hundred thousand CD34^+^ cells were cultured in erythroid differentiation media (EDM), which consists of IMDM (Fisher Scientific) supplemented with 15% FBS, 1% Bovine Albumin Fraction V (Fisher Scientific), 200 μg/ml holo-transferrin (Sigma-Aldrich), Insulin-Transferrin-Selenium (Fisher Scientific), and 2-mercaptoethanol (0.1 mM; SIGMA), supplemented with 50 ng/ml human stem cell factor (SCF), 6 IU/ml human EPO, and 10 ng/ml human interleukin-3. After 6 days culture, the cells were cultured in EDM supplemented with EPO and SCF for 4 more days.

### Chemical compound screening using Cellomics^TM^ ArrayScan®

Prior to the screening assay, dead cells in the culture were removed using Dead Cell Removal kit (Miltenyi Biotec) by following the manufacturer’s instruction. Ten thousand HiDEP cells in 100 μl of the complete medium per well were plated into 96-well tissue-culture flat-bottom plates. Two concentrations (0.5 μM and 10 μM) of chemical compounds dissolved in DMSO were added to the 96-well plates. Equivalent volumes of DMSO (0.005% and 0.1% in volume) were used as control for 0.5 μM and 10 μM, respectively. Cells were then cultured in 5% CO_2_ at 37 °C for 4 days.

After 4 days culture, cells were stained with 0.5 μM SYTO 16 (cell permeant nucleic acid dye) and 10 nM of SYTOX Red (non-permeant nucleic acid dye) (Invitrogen). Images of 50 fields within each well were captured using Cellomics^TM^ ArrayScan® VTT MCS Reader (Thermo Scientific) to measure the intensity of SYTO 16, SYTOX Red, and KuO in each cell and the collected data were analyzed on ArrayScan® VTI 700 Series. Enucleation efficiency was determined by the frequency of the cells that were negative for both dyes (representing no nucleus inside the cell) divided by the total number of cells expressing KuO and negative for SYTOX Red.

### Flow cytometry analysis

To quantify enucleation efficiency and cell viability, HiDEP treated with candidate chemical compounds were stained with SYTO 16 (Fisher Scientific) and 7AAD (Sigma-Aldrich). Enucleated cells were determined as cells negative for both SYTO 16 and 7AAD. For maturation analysis, cells were stained with anti-Human CD235a-A (GA-R2, BD), -CD71 (M-A712 BD), -Band3 (BIII 136, Fisher Scientific), and -CD49d (63-0499-42, Fisher Scientific) antibodies. Cells were analyzed on FACS LSRII (BD) and collected data were analyzed using FlowJo software (Tree Star).

### Cytospin and May–Grünwald–Giemsa staining

Cells were cytospun using Shandon Cytospin 3 (Block Scientific, Inc.) at 500 r.p.m. for 3 min. Air-dried slides were stained in May–Grünwald solution (Merck) for 5 min. After a brief wash with water, the slides were then stained in Giemsa solution (Histolabs) for 10 min. The slides were finally washed in distilled water and allowed to air dry before microscopic analysis. The number of enucleated cells/nucleated cells were manually counted from 50 to 70 fields in each cytospin slide.

### Time-lapse imaging

To capture time-lapse images of live HiDEP undergoing enucleation, Celldiscoverer 7 (Zeiss) was used. Cells were stained with Hoechst 33342 or NucRed Live 647 (ThermoFisher Scientific) to distinguish the nuclei. Thirty thousand cells per well were plated and indicated chemical compounds were added. Images were taken every 16 min for 48 h with ×20 magnification.

### Caspase-3 activity measurement

Caspase-3 activity was measured using Caspase-3 Colorimetric Activity Assay Kit (Sigma-Aldrich). Two-hundred thousand cells were incubated with DMSO or HDACi for 60 min and then enzymatic activity of Caspase-3 was measured by following the manufacturer’s instruction.

### RNA isolation and microarray analysis

FS (10 μM) or M344 (10 μM) was added and HiDEP were cultured for 24 h. Equivalent volume (0.1%) of DMSO was added as a control. Total RNA was extracted using RNeasy Kit (QIAGEN) by following the manufacturer’s instruction. RNA concentration and integrity were measured using Agilent RNA Pico Kit on a Bioanalyzer Instrument (Agilent Genomics). The microarray data are available at the GEO database under the accession number GSE138958.

### Quantitative reverse-transcriptase PCR

Total mRNA was extracted from HiDEP using RNeasy kit (QIAGEN) and cDNA were synthesized using SuperScript III reverse transcriptase (LifeTechnology). Real-time PCR reactions were performed on 7900 HT Fast Real-Time PCR System (Applied Biosystems). The following primers obtained from Applied Biosystems were used: HDAC1 (Mm02745760_g1), HDAC2 (Hs00231032_m1), HDAC3 (Hs00187320_m1), HDAC4 (Hs01041648_m1), HDAC5 (Hs00608351_m1), HDAC6 (Hs00997427_m1), HDAC7 (Hs01045864_m1), HDAC8 (Hs00954353_g1), HDAC9 (Hs01081558_m1), HDAC10 (Hs00368899_m1), HDAC11 (Hs00978031_g1), SPTA1 (Hs00162179_m1), SPTAN1 (Hs00949408_m1), KIF1B1 (Hs01114511_m1), KIF3A (Hs00199901_m1), KIF13B (Hs00209573_m1), KIF24 (Hs01592658_g1), SLC4A1 (Hs00978607_g1), SPTB (Hs01024093_m1), RHD (Hs00414315_m1), GYPC (Hs00242584_m1), KLF1 (Hs00610592_m1), and HPRT1 (Hs02800695_m1). All ΔCt values were normalized using *HPRT1*.

### Western blotting

Proteins were extracted from one million cells after 2.5 h of treatment with DMSO or HDACi using RIPA buffer supplemented with 1 M NaF, 10 mM phenylmethylsulfonyl fluoride, and a protease inhibitor cocktail. For detection of specific acetylation of histone H3 and H4, the following antibodies were used: Acetyl-Histone H3 Antibody Sampler kit #9927 (Cell Signaling), anti-H3K23 (D6Y7M, Cell signaling), -H4K5 (EP1000Y, Abcam), -H4K8 (EP1002Y, Abcam), -H4K12 (Abcam), -H4K16 (EPR1004, Abcam), and -Histone H4 (Abcam).

### HDAC activity measurement

Enzymatic activities of HDAC were measured using HDAC Kinetic Assay Kit (HDAC1, HDAC2, HDAC3, BPS Bioscience), HDAC5 Fluorogenic Kit (BPS Biosciences), and HDAC6 Activity Assay Kit (BioVision) by following the manufacturer’s instruction. Enzymatic activity was measured in 200,000 cells after 1 h treatment with DMSO or HDACi.

### CRISPR activation

HiDEPs were first transduced with lentiviruses expressing dCas9-VPR (Dharmacon) and transduced cells were sorted out based on green fluorescent protein (GFP) expression, which represents dCas9-VPR expression. HiDEP-dCas9 were then transduced with lentiviruses containing sgRNA targeting *SPTA1* (VSGH11888–247240446, Dharmacon). One week after transduction, cells were single cell-sorted and each clone was grown separately.

### Lentiviral overexpression of *KLF1*

Human *KLF1* gene was amplified from cDNA of HiDEP using following primers: Fwd, 5′-TACACCGGTGAGAGTTCACGAGGCAGCCGAGGAA-3′; Rev, 5′- TACGGCCGTCAAAGGTGGCGCTTCATGTG-3′. The amplified gene was then inserted to pSFFV-MCS-IRES2-GFP vector. Lentivirus was produced in HEK293T cells and collected lentiviral particles were concentrated using ultracentrifuge. HiDEP was transduced with the virus and GFP^+^ cells were sorted using FACS AriaIII (BD).

### Statistics and reproducibility

Statistical methods determining significance are described in each figure legend. All statistical analyses were performed on Prism (Graphpad). To repeat experiments, all samples were always newly prepared or conditioned.

### Reporting summary

Further information on research design is available in the Nature Research Reporting Summary linked to this article.

## Supplementary information

Supplementary Materials

Description of Supplementary Files

Supplementary Movie 1

Supplementary Movie 2

Supplementary Data 1

Reporting Summary

## Data Availability

The microarray data are available at the GEO database under the accession number GSE138958. The source data for all graphs and charts are given in Supplementary Data 1 and any remaining information can be obtained from the corresponding author upon reasonable request.
