# Supplementary material for: Identification of potential chemical compounds enhancing generation of enucleated cells from immortalized human erythroid cell lines
Source: Commun Biol. 2021 Jun 3;4:677. doi: 10.1038/s42003-021-02202-1 (PMC8175573; doi:10.1038/s42003-021-02202-1)
Supplement: Supplementary file 1 — Supplementary Materials [file 42003_2021_2202_MOESM1_ESM.pdf]

Supplementary Figures

Identification of potential chemical compounds enhancing generation of enucleated cells from immortalized human erythroid cell lines

Svetlana Soboleva, Ryo Kurita, Fredrik Ek, Hugo Åkerstrand, Rita Silvério-Alves, Roger Olsson, Yukio Nakamura, and Kenichi Miharada

Supplementary Figure S1

Supplementary Figure S2

Supplementary Figure S3

Supplementary Figure S4

Supplementary Figure S5

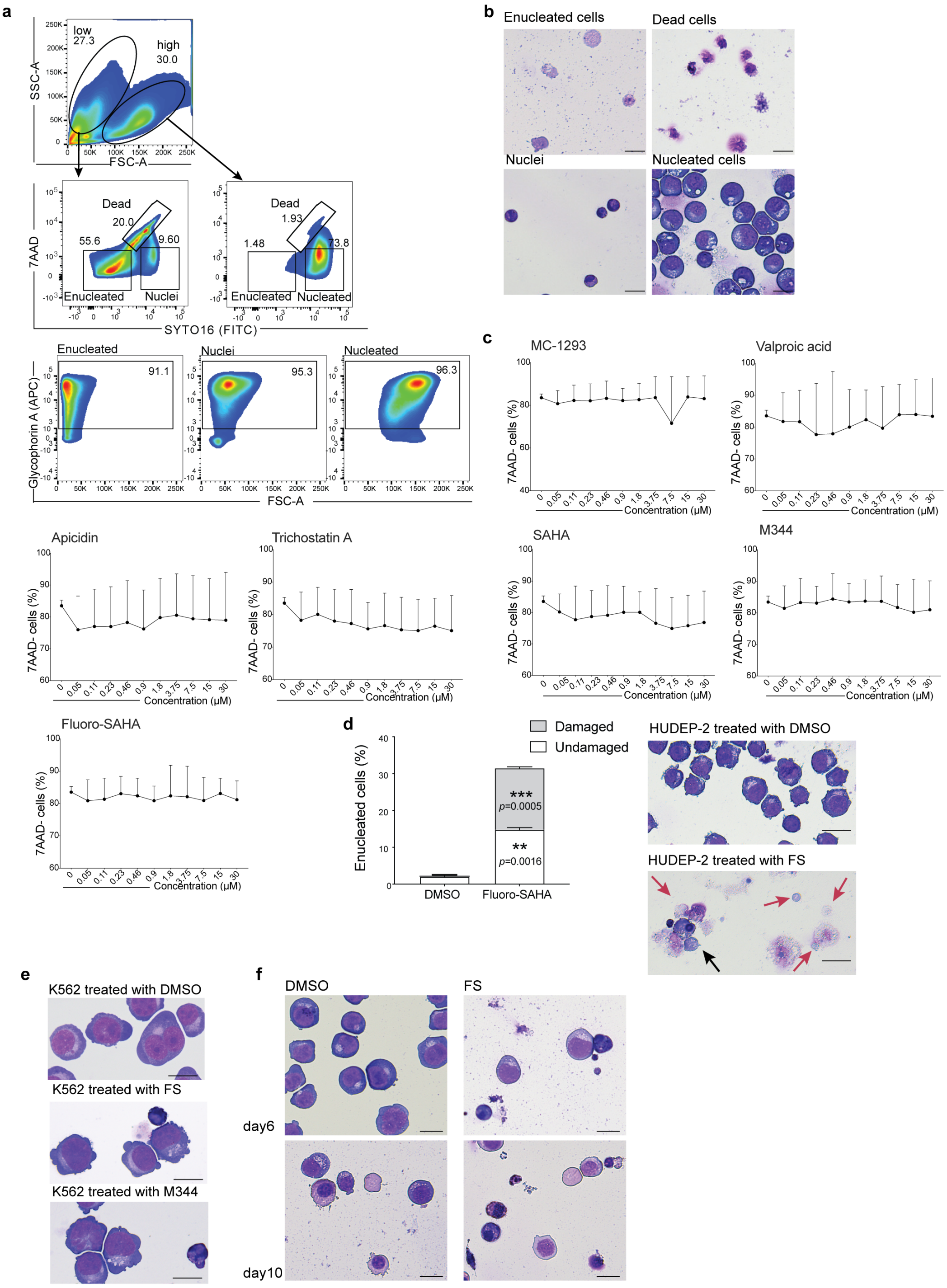

Supplementary Figure 1. Effect of HDACi on viability of HiDEP and other cell lines

a Flow cytometry analysis of HDACi-treated HiDEP. Cells were subdivided to FSC^{high} and FSC^{low} populations, and then based on the intensity of SYTO16 and

7AAD the cells were categorized to $FSC^{low}SYTO16^{-}7AAD^{-}$ (enucleated cells), $FSC^{low}SYTO16^{+}7AAD^{-}$ (nuclei), $FSC^{high}SYTO16^{+}7AAD^{-}$ (nucleated cells) and $SYTO16^{+}7AAD^{+}$ (dead cells and disrupted nuclei) subpopulations. In each subpopulation, Glycophorin-A expression was analyzed. **b** Representative May-Grünwald Giemsa staining of different fractions sorted from HiDEP treated with Fluoro-SAHA. **c** Viability change of HiDEP treated with selected HDACi. Frequencies of $7AAD^{+}$ cells 24 hours after treatment with different concentrations of HDACi are shown. Mean ± SD, n=3. Significance was determined using one-way ANOVA with Dunnett's multiple comparisons test. **d** Frequency of enucleated cells from HUDEP-2 treated with DMSO or Fluoro-SAHA. Undamaged and damaged enucleating cells were manually counted on 45-50 fields from cytospin slides. Mean values of enucleation frequency in 3 slides (left) and representative May-Grünwald Giemsa staining of cytospin slides (right) are shown. Black arrow: undamaged cells, red arrows: damaged cells. Mean ± SD, n=3. Significance was determined using paired *t* test. **e** Representative May-Grünwald Giemsa staining of K562 treated with DMSO or Fluoro-SAHA or M344. **f** Representative May-Grünwald Giemsa staining of Glycophorin-A^{+} cells differentiated from hUCB-derived $CD34^{+}$ cells treated with DMSO or Fluoro-SAHA on day 6 and day 10.

$^{**}p < 0.01$, $^{***}p < 0.001$.

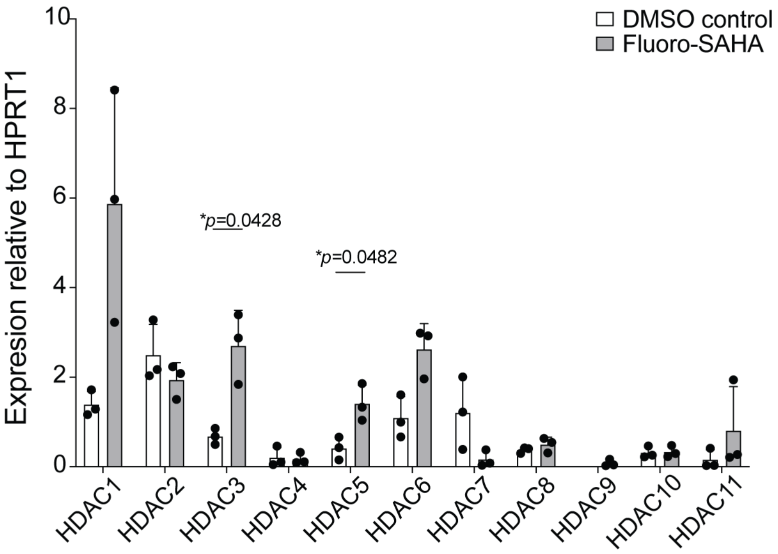

Supplementary Figure 2. Expression levels of HDAC genes in HiDEP.

qRT-PCR analysis for the expression of HDAC genes in HiDEP treated with DMSO or Fluoro-SAHA. The expression levels are normalized to *HPRT1*. Mean ± SD, n=3. Significance was determined using paired *t* test. $*p < 0.05$

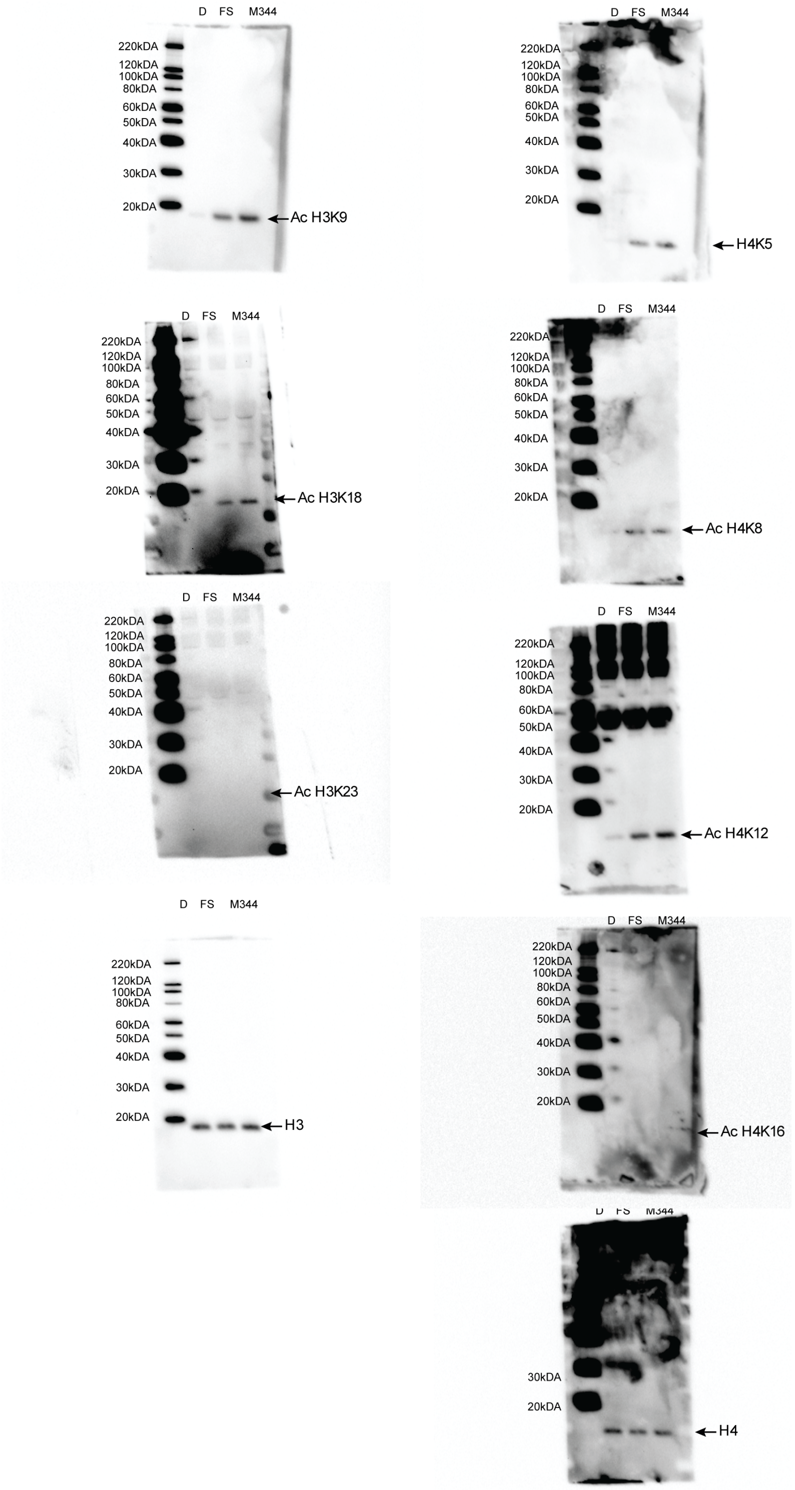

Supplementary Figure 3. Western blot analysis of acetylation states of histone H3 and H4 in HiDEP treated with FS or M344.

Full pictures of each blot are displayed.

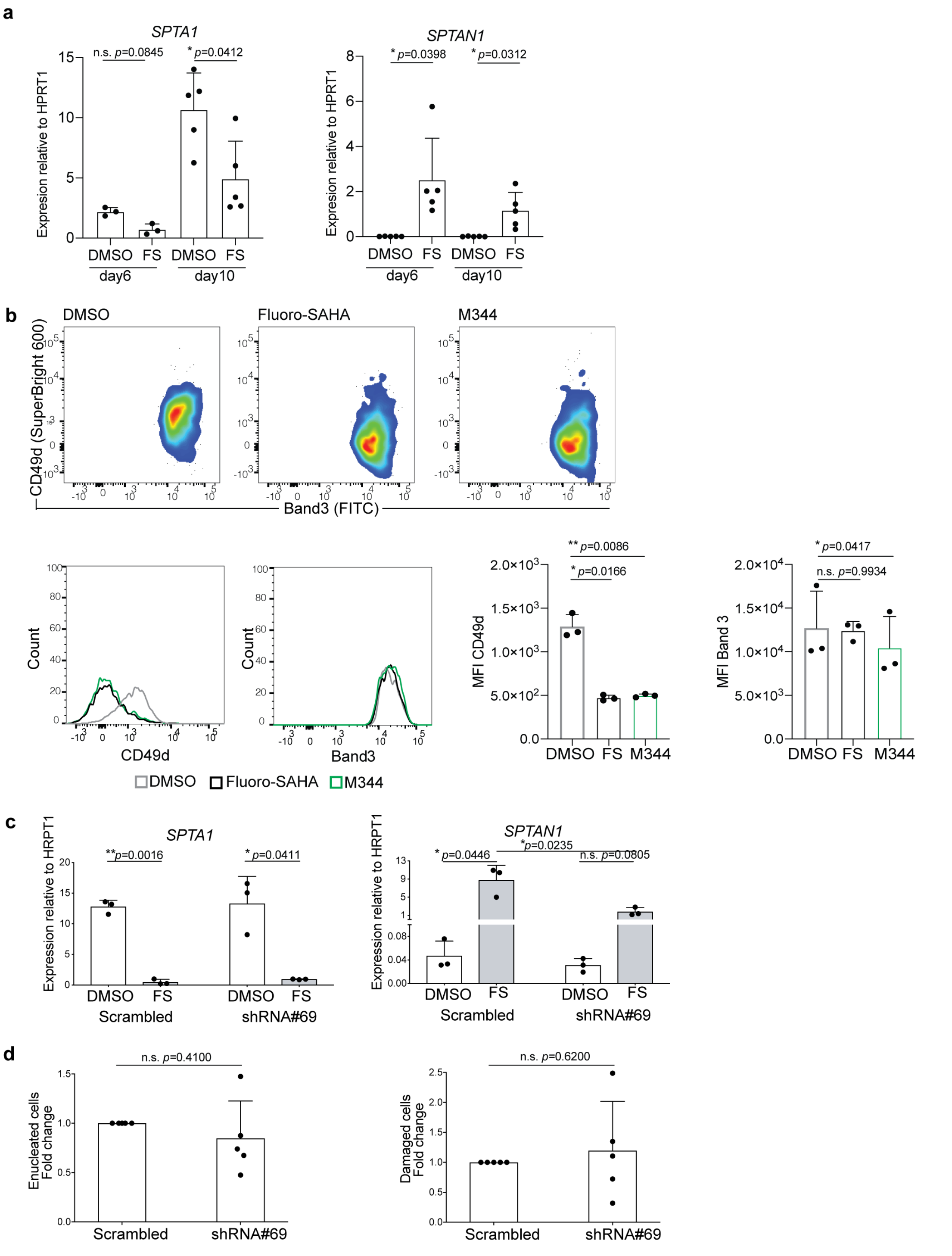

Supplementary Figure 4. Impact of *SPTAN1* knock-down on enucleation efficiency and effect of *SPTA1* enhancement on the cell surface phenotype. **a** qRT-PCR analysis for the expression of *SPTA1* and *SPTAN1* in Glycophorin-A$^+$ cells differentiated from hUCB-derived CD34$^+$ cells with or without FS

treatment. The expression levels are normalized to *HPRT1*. Mean ± SD, n=5. Significance was determined using paired *t* test. **b** Effect of SPTA1 activation using CRISPRa on cell surface phenotype. (Top) Representative FACS profiles of CD49d and Band3 expression on SPTA1-activated HiDEP (clone #5) treated with DMSO, Fluoro-SAHA or M344. (Lower) Histogram (left) and MFI comparison (right) of CD49d and Band3 expression. Mean ± SD, n=3. Significance was determined using one-way ANOVA with Dunnett's multiple comparisons test. **c** qRT-PCR analysis for the expression of *SPTA1* and *SPTAN1* in HiDEP cells expressing shRNA against *SPTAN1* (shSPTAN1) with or without FS treatment. The expression levels are normalized to *HPRT1*. Mean ± SD, n=3. Significance was determined using paired *t* test. **d** Frequencies of undamaged (intact) enucleated cells and damaged enucleated cells in HiDEP-shSPTAN1 (#69) with or without FS. Mean ± SD, n=5. Significance was determined using paired *t* test. *$p < 0.05$, **$p < 0.01$.

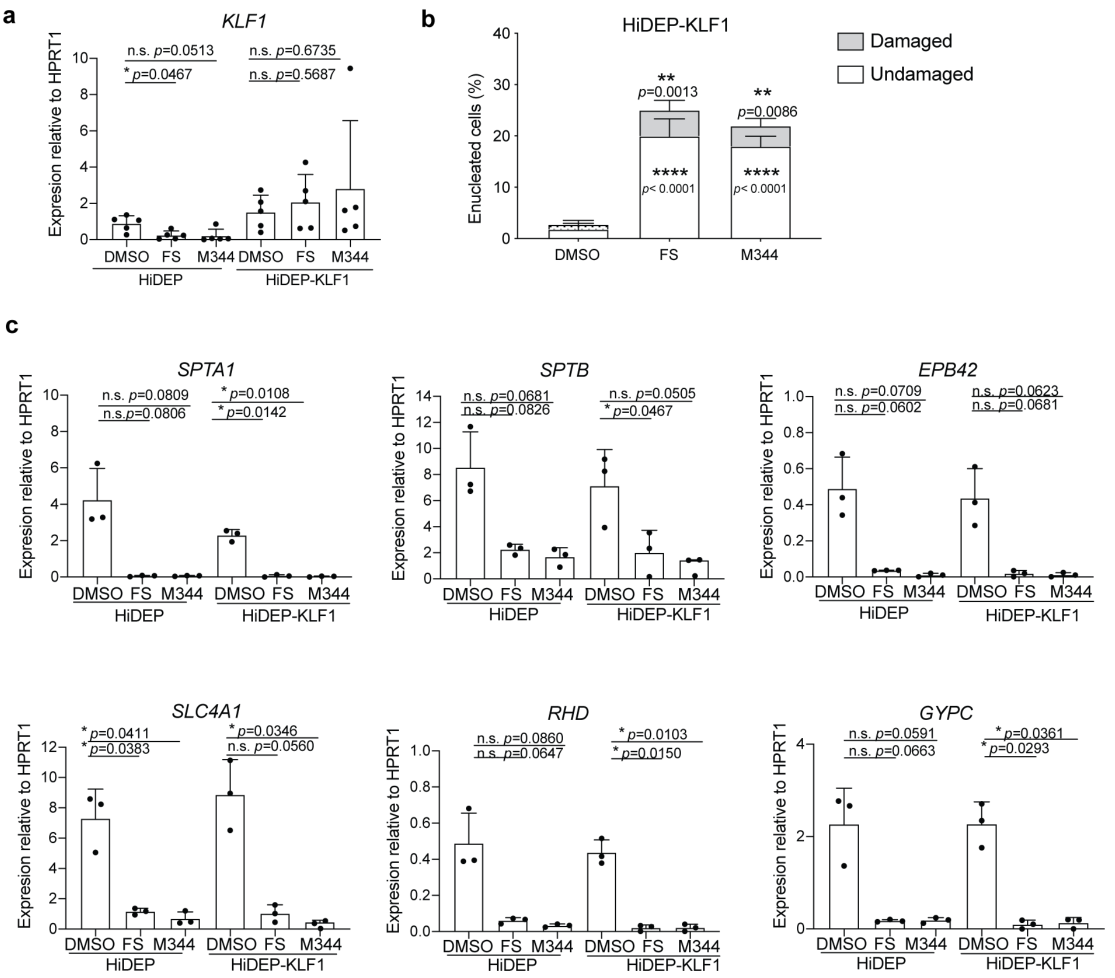

Supplementary Figure 5. Effect of *KLF1* overexpression on enucleation efficiency and expression levels of cell membrane protein genes.

a qRT-PCR analysis for the expression of *KLF1* in HiDEP overexpressing *KLF1* (HiDEP-KLF1) with or without FS/M344 treatment. The expression levels are normalized to *HPRT1*. Mean ± SD, n=5. Significance was determined using one-way ANOVA with Dunnett's multiple comparisons test. **b** Frequencies of enucleated cells in HiDEP-KLF1 with or without FS/M344 treatment. Mean ± SD, n=7. Significance was determined using one-way ANOVA with Dunnett's multiple comparisons test. **c** qRT-PCR analysis for the expression of representative cell membrane protein genes in HiDEP-KLF1 with or without FS/M344 treatment. The

expression levels are normalized to *HPRT1*. Mean ± SD, n=3. Significance was determined using one-way ANOVA with Dunnett's multiple comparisons test. *$p < 0.05$, **$p < 0.01$, ***$p < 0.001$, ****$p < 0.0001$.
