# Supplementary material for: Identification of potential chemical compounds enhancing generation of enucleated cells from immortalized human erythroid cell lines
Source: Commun Biol. 2021 Jun 3;4:677. doi: 10.1038/s42003-021-02202-1 (PMC8175573; doi:10.1038/s42003-021-02202-1)
Supplement: Supplementary file 2 — Description of Supplementary Files [file 42003_2021_2202_MOESM2_ESM.pdf]

Description of Additional Supplementary Files

File name: Supplementary Movie 1.

Description: A representative time-lapse video of HiDEP treated with Fluoro-SAHA undergoing standard enucleation. Images were captured every 6 minutes. Yellow, Kusabira Orange; Red, NucRed Live 647.

File name: Supplementary Movie 2.

Description: A representative time-lapse video of HiDEP cells treated with Fluoro-SAHA under going rupture-style enucleation. Images were captured every 6 minutes. Yellow, Kusabira Orange; Red, NucRed Live 647.

File name: Supplementary Data 1

Description: The source data for all graphs and charts
