# Supplementary material for: Identification of potential chemical compounds enhancing generation of enucleated cells from immortalized human erythroid cell lines
Source: Commun Biol. 2021 Jun 3;4:677. doi: 10.1038/s42003-021-02202-1 (PMC8175573; doi:10.1038/s42003-021-02202-1)
Supplement: Supplementary file 6 — Reporting Summary [file 42003_2021_2202_MOESM6_ESM.pdf]

nature research

Corresponding author(s): Kenichi Miharada

Last updated by author(s): Apr 12, 2021

Reporting Summary

Nature Research wishes to improve the reproducibility of the work that we publish. This form provides structure for consistency and transparency in reporting. For further information on Nature Research policies, see our Editorial Policies and the Editorial Policy Checklist.

Statistics

For all statistical analyses, confirm that the following items are present in the figure legend, table legend, main text, or Methods section.

n/a	Confirmed	
☐	☒	The exact sample size (*n*) for each experimental group/condition, given as a discrete number and unit of measurement
☐	☒	A statement on whether measurements were taken from distinct samples or whether the same sample was measured repeatedly
☐	☒	The statistical test(s) used AND whether they are one- or two-sided<br>*Only common tests should be described solely by name; describe more complex techniques in the Methods section.*
☒	☐	A description of all covariates tested
☒	☐	A description of any assumptions or corrections, such as tests of normality and adjustment for multiple comparisons
☐	☒	A full description of the statistical parameters including central tendency (e.g. means) or other basic estimates (e.g. regression coefficient) AND variation (e.g. standard deviation) or associated estimates of uncertainty (e.g. confidence intervals)
☐	☒	For null hypothesis testing, the test statistic (e.g. *F*, *t*, *r*) with confidence intervals, effect sizes, degrees of freedom and *P* value noted<br>*Give P values as exact values whenever suitable.*
☒	☐	For Bayesian analysis, information on the choice of priors and Markov chain Monte Carlo settings
☒	☐	For hierarchical and complex designs, identification of the appropriate level for tests and full reporting of outcomes
☒	☐	Estimates of effect sizes (e.g. Cohen's *d*, Pearson's *r*), indicating how they were calculated

Our web collection on statistics for biologists contains articles on many of the points above.

Software and code

Policy information about availability of computer code

Data collection	no
Data analysis	Microarray data was analyzed in R (version 3.5.0) using R studios (version 1.2.5019), heatmaps were generated using the pheatmap package (version 1.0.10). The data was also analyzed using Gene Set Enrichment Analysis (GSEA, version 4.0.0), and the results were redrawn using the replotGSEA function from the Rtoolbox package. All statistical analyses were done using Prism (version 8, GraphPad).

For manuscripts utilizing custom algorithms or software that are central to the research but not yet described in published literature, software must be made available to editors and reviewers. We strongly encourage code deposition in a community repository (e.g. GitHub). See the Nature Research guidelines for submitting code & software for further information.

Data

Policy information about availability of data

All manuscripts must include a data availability statement. This statement should provide the following information, where applicable:

- Accession codes, unique identifiers, or web links for publicly available datasets
- A list of figures that have associated raw data
- A description of any restrictions on data availability

The microarray data (Figure 6) are available at the GEO database under the accession number GSE138958

Field-specific reporting

Please select the one below that is the best fit for your research. If you are not sure, read the appropriate sections before making your selection.

- [x] Life sciences
- [ ] Behavioural & social sciences
- [ ] Ecological, evolutionary & environmental sciences

For a reference copy of the document with all sections, see nature.com/documents/nr-reporting-summary-flat.pdf

Life sciences study design

All studies must disclose on these points even when the disclosure is negative.

Sample size	No prospective sample-size calculations were performed. Sample size was determined retrospectively to be adequate on the basis of the consistency and magnitude
Data exclusions	No data was excluded
Replication	All experiments were repeated more than three times to verify the results. For CRISPR and shRNA-related experiments multiple clones (both successful and failed editing/down-regulation were expected) were included in the assay in order to confirm the consistency.
Randomization	The chemical compound screening was performed using pre-deposited plates. In order to false signals based on the "edge effect" in which specific loci of wells in plastic plates are affected by unintended factors (e.g. evaporation and reflection of fluorescence), control assay were performed to compare fluorescent signals between random positions.
Blinding	Scoring of enucleated cells on cytospin slides were performed without showing the sample labelling, as a blind test.

Reporting for specific materials, systems and methods

We require information from authors about some types of materials, experimental systems and methods used in many studies. Here, indicate whether each material, system or method listed is relevant to your study. If you are not sure if a list item applies to your research, read the appropriate section before selecting a response.

Materials & experimental systems

n/a	Involved in the study	
[ ]	[x]	Antibodies
[ ]	[x]	Eukaryotic cell lines
[x]	[ ]	Palaeontology and archaeology
[x]	[ ]	Animals and other organisms
[x]	[ ]	Human research participants
[x]	[ ]	Clinical data
[x]	[ ]	Dual use research of concern

Methods

n/a	Involved in the study	
[x]	[ ]	ChIP-seq
[ ]	[x]	Flow cytometry
[x]	[ ]	MRI-based neuroimaging

Antibodies

Antibodies used	Anti-Human 235a (Clone GA-R2, cat.# 551336, BD); Anti-Human CD71 (Clone M-A712, cat.# 562995, BD); Anti-Human Band3 (Clone BIII 136, Fisher Scientific); Anti-Human CD49d (Clone 9F-10, cat.# 63-0499-42 , Fisher Scientific); Acetyl-Histone H3 Antibody Sampler kit (cat. #9927, Cell Signaling); anti-Human H3K23 (Clone D6Y7M, cat.# 14932S, Cell signaling); anti-Human H4K5 (Clone EP1000Y, cat.# ab51997, Abcam); anti-Human H4K8 (Clone EP1002Y, cat.# ab45166, Abcam); anti-Human H4K12 (cat.# ab46983, Abcam); anti-Human H4K16 (Clone EPR1004, cat.# ab109463, Abcam); anti-Human Histone H4 (cat.# ab10158, Abcam).
Validation	All antibodies against cell surface molecules were first validated using mononuclear cells isolated from human umbilical cord blood, according to the original report (Hu et al., Blood, 2013). Antibodies against histone and specific acetylation are described in the previous study (Jayapal et al., J Biol Chem, 2010).

Eukaryotic cell lines

Policy information about cell lines

Cell line source(s)	HiDEP-1, HUDEP-2 and K562 cell lines were obtained from the RIKEN Cell Bank (Tsukuba, Ibaraki, Japan).
Authentication	Cellular quality and characteristics are guaranteed by RIKEN Cell Bank.
Mycoplasma contamination	Negativity of mycoplasma has been confirmed by RIKEN Cell Bank.

Commonly misidentified lines (See ICLAC register)	The original characteristics of K562 including its differentiation potential has been guaranteed by RIKEN Cell Bank.

Flow Cytometry

Plots

Confirm that:

☒ The axis labels state the marker and fluorochrome used (e.g. CD4-FITC).

☒ The axis scales are clearly visible. Include numbers along axes only for bottom left plot of group (a 'group' is an analysis of identical markers).

☒ All plots are contour plots with outliers or pseudocolor plots.

☒ A numerical value for number of cells or percentage (with statistics) is provided.

Methodology

Sample preparation	All studies were done using cell lines.
Instrument	A high-throughput analysis of fluorescent signals was done using Cellomics ArrayScan® VTT MCS Reader (Thermo Scientific). Flow cytometry analyses were done on FACS LSRII (BD). Time-lapse images were captured using Celldiscoverer 7 (Zeiss). qRT-PCR analyses were done using 7900HT Fast Real-Time PCR System (Applied biosystems).
Software	The collected fluorescent images were analyzed on ArrayScan® VTI 700 Series (Thermo Scientific). Flow cytometry data were analyzed using FlowJo software (Tree Star). All statistical analyses were done using Prism (GraphPad).
Cell population abundance	We did not sort specific population, with an exception of GFP+ cells after transduction of dCas9-VRP expressing lentivirus. GFP positivity of the sorted cells was confirmed after their proliferation.
Gating strategy	For 7AAD and SYTO16 staining unstained control was used to determine the gates.

☒ Tick this box to confirm that a figure exemplifying the gating strategy is provided in the Supplementary Information.
